# Causal network analysis of omics data using prior knowledge databases

**DOI:** 10.1093/bib/bbaf654

**Published:** 2025-12-05

**Authors:** Gleb Svinin, Enrico Glaab

**Affiliations:** Biomedical Data Science Group, Luxembourg Centre for Systems Biomedicine (LCSB), University of Luxembourg, 7, avenue des Hauts Fourneaux, L-4362 Esch-sur-Alzette, Luxembourg; Biomedical Data Science Group, Luxembourg Centre for Systems Biomedicine (LCSB), University of Luxembourg, 7, avenue des Hauts Fourneaux, L-4362 Esch-sur-Alzette, Luxembourg

**Keywords:** molecular networks, prior knowledge, systems biology, bioinformatics workflows, network analysis, causal reasoning

## Abstract

Identifying causal relationships in omics data is essential for understanding underlying biological processes. However, detecting these relationships remains challenging due to the complexity of molecular networks and observational data limitations. To guide researchers, we conducted a systematic literature review of data-driven causal omics analysis methods that use structured prior knowledge from regulatory and interaction databases. We grouped methods into three approaches based on the extent of prior knowledge integration: regulon-level (direct regulator–target links, straightforward interpretation, but with the risk of oversimplification), flow-level (multi-step propagation from regulators to targets, broader mechanism explanation, but lacking uncertainty modeling), and network-level (system-wide interactions and crosstalk, most comprehensive, but with increased computational complexity and requiring particularly careful interpretation). These methods have demonstrated utility across diverse applications, including identification of therapeutic targets in acute myeloid leukemia, elucidation of mechanisms in IgA nephropathy, and detection of regulatory perturbations in Alzheimer’s disease. We discuss the strengths, limitations, and representative use cases of each approach, and address general limitations and outline future research directions. This review serves as a practical guide for the entire analysis process, from selecting prior knowledge databases (PKDBs) to choosing and applying causal analysis methods for different research questions.

## Introduction

Understanding causal relationships in cellular processes is one of the main goals in systems biology. Although statistical analysis methods are effective at identifying differential patterns in large-scale biological data, they often fail to reveal the underlying mechanisms. These methods are generally unable to distinguish between correlation and causation and do not provide insights into the upstream regulatory factors that could explain observed alterations.

To address these limitations, bioinformatics approaches have been developed for causal molecular network analysis. These methods integrate experimental data with prior biological knowledge to provide mechanistic data interpretations. By combining data- and knowledge-driven reasoning, causal network analysis identifies upstream regulators and perturbations likely responsible for the measured changes in gene or protein activities. In a disease context, studying these causal mechanisms can improve understanding of normal homeostasis and pathological processes, and potentially highlight new candidate drug targets for preclinical intervention studies [1].

A key aspect of causal network analysis involves using prior knowledge databases (PKDBs), which contain information about interactions between biological entities. In recent years, the quantity and quality of available prior knowledge has grown rapidly, driven by large-scale curation efforts [2]. This progress has provided a solid foundation for developing computational methods that rely on prior knowledge for both associative [3] and causal analyses.

Diverse causal network analysis methods have been proposed, differing significantly in how they integrate prior knowledge, use experimental data, and the outputs they produce. As molecular datasets grow in size and complexity, causal network analysis is playing an increasingly important role in identifying disease mechanisms and supporting translational research.

While previous reviews have addressed causal analysis of molecular data, their focus differs from that of the present work. For example, Kelly *et al.* [4] reviewed methods for *inferring* causal links and *de novo* network construction from observational data, independent of prior knowledge (e.g. Mendelian randomization, Bayesian causal inference, and time-series causal inference). Other reviews have focused on specific applications, such as drug discovery or repurposing, rather than on the broader class of approaches combining experimental data with structured biological knowledge [5, 6]. Most relevant to our work, Nguyen [7] and Garrido-Rodríguez [8] explored data-driven methods that incorporate prior knowledge; however, they focused on comparing altered pathways and subnetworks rather than identifying and ranking key causal upstream regulators, which is the primary focus of this review.

**Figure 1:**
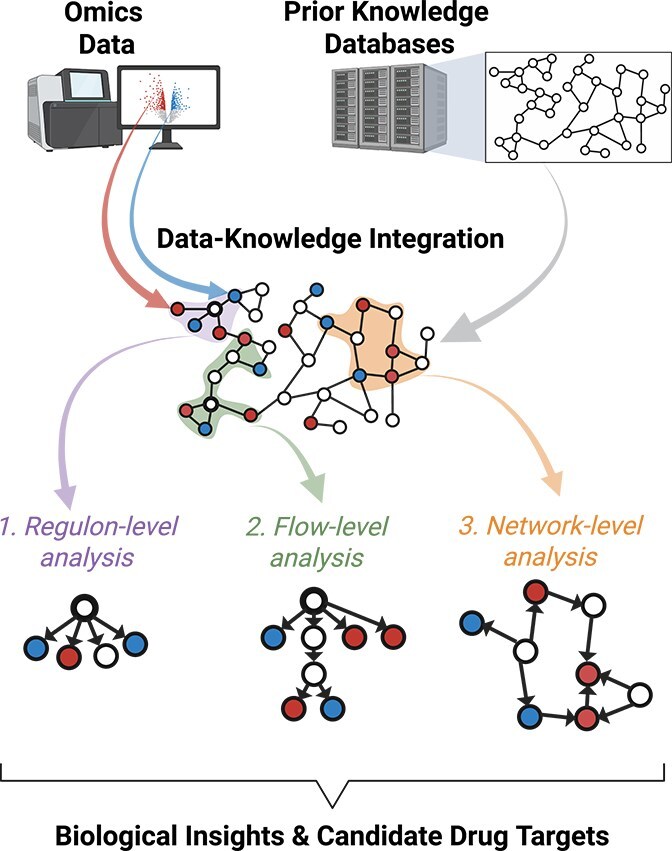
Overview of the workflow for causal network analysis of omics data. Experimental omics data and prior knowledge databases are integrated through three complementary analytical approaches that differ in scope: regulon-level analysis examines individual regulators and their direct targets (left network, highlighted node shows putative regulator); flow-level analysis traces multi-step signaling cascades from upstream sources to downstream effectors (middle network, highlighted node shows upstream regulator); and network-level analysis considers system-wide regulatory interactions and crosstalk (right network). Arrows represent causal relations directed from source to target. Node colors indicate experimental states: upregulated (blue), downregulated (red), or not affected (white). These three methodological categories, detailed in the Methods section, provide increasingly comprehensive but computationally complex approaches to identifying causal mechanisms underlying observed molecular changes. Alt text: Diagram showing three network analysis approaches of increasing complexity: regulon-level analysis with a single highlighted regulator node, flow-level analysis with highlighted upstream regulator, and network-level analysis showing system-wide interactions.

To our knowledge, this is the first comprehensive review centered specifically on data-driven causal analysis methods that incorporate structured prior knowledge. We propose that these methods represent a category of their own, with growing importance in computational systems biology. A particular benefit of causal network analyses is their potential to generate biologically meaningful insights from datasets with relatively small sample sizes, a common situation for many biomedical studies. Causal network analysis is still feasible in this scenario because prior data on known interactions is exploited, helping to narrow down the search space of possible causal mechanisms. Through this review, we aim to provide practical recommendations for conducting causal network analyses, addressing each step of the process from prior knowledge database selection to interpretation of the results (see Fig. 1).

## Methods

We conducted a systematic literature review to identify and evaluate computational methods for causal network analysis of omics data using prior biological knowledge. This review follows established guidelines for systematic reviews, specifically adhering to the scoping review methodology outlined by the Joanna Briggs Institute (JBI) [9].

### Literature search strategy

We searched PubMed, Web of Science, and IEEE Xplore databases (last search date: April 2025) for articles describing computational methods that integrate experimental omics data with prior knowledge networks (PKNs) to infer causal regulatory mechanisms. Our search strategy employed a multistage keyword-based approach using four categories of search terms: (i) “Causal analysis” (causal reasoning, upstream regulator), (ii) “Network analysis” (network contextualization, pathway analysis), (iii) “Prior knowledge” (prior knowledge database, gene regulatory network), and (iv) “Omics data” (transcriptomics, proteomics). Articles were required to contain at least one relevant search term from each category. We supplemented database searches with manual screening of reference lists from key review articles. We restricted inclusion to peer-reviewed articles published from January 2010 to April 2025 in English.

### Eligibility criteria and data extraction

We included articles describing computational methods that: (i) integrate experimental omics data with structured prior biological knowledge, (ii) aim to identify candidate upstream regulators or causal mechanisms, (iii) provide algorithmic descriptions sufficient for implementation, and (iv) demonstrate application to real biological datasets. We excluded methods that: (i) focus solely on *de novo* network inference; (ii) describe purely correlation-based approaches; (iii) strictly require experimental perturbation data as primary input; or (iv) lack complete mathematical formulations, pseudocode, step-by-step algorithmic descriptions, or validation. Two reviewers independently checked articles for eligibility, and for each included method, we extracted information on its mathematical formulation, data requirements, PKDB compatibility, software availability, and biological applications.

Methods were categorized based on their extent of prior knowledge integration: regulon-level (direct regulator-target relationships), flow-level (multi-step regulatory cascades), and network-level (system-wide regulatory analysis; see detailed discussion of each category below). This classification emerged iteratively during the review process. We synthesized the literature by conducting a comparative analysis of methodological approaches to provide practical guidance for method selection.

## Data resources

### Prior knowledge databases

A PKDB is any structured resource that catalogs directed molecular or gene regulatory interactions, specifying the source, target, and the type of effect (e.g. activating or inhibiting). For the purposes of this review, PKDBs are assumed to contain directed interactions in a triplet format: source, target, and sign of the interaction. While not all pathway databases explicitly follow this model (e.g. KEGG [10], Reactome [11], PANTHER [12]), causal information is sometimes embedded within their biological process descriptions. We focus on PKDBs that are directly used in the reviewed methods (see Table 1), while a broader overview of available PKDBs has been presented by Touré *et al.* [13].

**Table 1:** Overview of prior knowledge databases for causal network analysis.

**Name**	**Specific nodes/bioentities** ^a^	**Interaction signs**	**Weighted edges**	**Accessibility**	**Ref**
SIGNOR 3.0	Phenotype, stimulus, antibody	All signed	No	Public	[14]
GeneGo MetaCore™	Kinase, phosphatase, enzyme DNA, G beta/gamma, G alpha, inorganic ion, ligand-gated ion channel	Signed and unsigned	No	Commercial	RRID: SCR_008125
QIAGEN Biomedical Knowledge Base - Human Derived (QBKB-HD)	Cytokine, disease, drug, function, group, canonical pathway, transcription regulator, translation regulator	Signed and unsigned	Yes	Commercial	RRID: SCR_008117
PhosphoSitePlus (PPP)	Proteins, kinases, phosphosites	Signed and unsigned	No	Licence upon individual conditions	[15]

This table compares three databases (SIGNOR, MetaCore and QBKB-HD) across four dimensions: their supported “Specific nodes / bioentities” (the term “bioentities” refer to biological entities such as genes, proteins, or metabolites, “specific” refers to terms not shared across all three databases), “Interaction signs” (whether directed relationships are signed or unsigned), “Weighted edges” (quantitative relationship strength indicators), and “Accessibility” (public, commercial, other), with supporting references in the “Ref” column

^a^SIGNOR, MetaCore, and QBKB-HD all include the following shared entity types: proteins, chemicals, complexes, and microRNAs. PPP is a specialized resource for prior knowledge on phosphoproteomics; we refer to its datasets on kinase-substrate interactions and regulatory sites.

Nodes and interactions in PKDBs provide two main types of information: the type of nodes covered (e.g. genes, proteins, drugs), which pre-determines the type of experimental data that can be mapped; and the type of signaling and regulatory interactions. Commonly used resources like GeneGo MetaCoreTM and QIAGEN Biomedical Knowledge Base - Human Derived (QBKB-HD) provide highly granular protein labels and drug nodes. The SIGNOR database [14], in turn, covers molecular phenotype nodes such as proliferation and apoptosis. Another essential information source is the sign of interactions (+ for activation, − for inhibition, and, sometimes, 0 for unknown), which defines rules for signal propagation. Some databases also assign weights to edges, reflecting the confidence of the evidence. These characteristics vary significantly and should be carefully considered when selecting a resource.

### Omics data resources

Causal network analysis is typically used to investigate omics data comparing different conditions, such as healthy versus diseased states. Most methods require a list of altered bioentities (e.g. genes or proteins) identified through differential analysis. At a minimum, this list should include identifiers and the direction of change (up- or down-regulated). Some methods also support quantitative information, such as log fold changes or *P*-values. Omics data can be derived from new experiments or from public repositories (see Table 2). Incorporating public data offers several advantages, including validating findings across multiple datasets and increasing statistical power [16]. When using public data, researchers should consider factors such as experimental design compatibility, batch effects, and platform differences [17]. Preprocessing steps like normalization and batch correction are essential to ensure data compatibility [18, 19]. In this review, we assume that experimental data has been preprocessed to produce a list of altered bioentities suitable for analysis.

**Table 2:** Key public resources for omics data in biomedical research.

**Omics type**	**Resource**	**URL / References**
**Transcriptomics**	Gene Expression Omnibus (GEO), ArrayExpress	www.ncbi.nlm.nih.gov/geo/ [20]; www.ebi.ac.uk/arrayexpress [21]
**Genomics**	dbGaP Database, NCBI Sequence Read Archive (SRA), European Nucleotide Archive (ENA)	www.ncbi.nlm.nih.gov/gap [22]; www.ncbi.nlm.nih.gov/sra [23]; www.ebi.ac.uk/ena [24]
**Proteomics**	EBI PRIDE, ProteomeXchange, MassIVE, PeptideAtlas	www.ebi.ac.uk/pride/ [25]; www.proteomexchange.org [26]; https://massive.ucsd.edu; www.peptideatlas.org [27]
**Metabolomics**	MetaboLights, Metabolomics Workbench	www.ebi.ac.uk/metabolights [28]; www.metabolomicsworkbench.org [29]
**Epigenomics**	IHEC Portal, MethBase	http://epigenomesportal.ca/ihec/ [30]; https://smithlabresearch.org/software/methbase/ [31]

This table summarizes major public repositories organized by omics data type, providing access to standardized datasets that can be used for causal network analysis either independently or in combination with private data.

### Filtering of prior knowledge databases

The content of a selected PKDB must be filtered before causal analysis. This is necessary for several reasons: contextual relevance (interactions from unrelated tissues may introduce noise), heterogeneity of bioentities (many entities may not be relevant to the omics data studied), and varying confidence levels (retaining only high-confidence interactions improves robustness). In addition, PKDBs carry systematic curation/literature biases: well-studied areas like cancer and immunology are often overrepresented, while rare diseases and some tissues or processes are underrepresented [32]. This skew can inflate the support for regulators in heavily curated domains, so results should be interpreted with that imbalance in mind.

Most commercial databases (like MetaCore and QBKB-HD) cannot be downloaded in full but instead return relevant bioentities and interactions based on predefined criteria, a process called the “connection strategy.” For instance, one might retrieve only direct interactions between a provided list of bioentities. For databases that can be downloaded entirely, connection strategies remain valuable for reducing computational complexity. Open-source tools like NeKo by Ruscone *et al.* [33] support flexible network assembly for this purpose. Once preprocessed, a PKDB is referred to as a PKN.

## Causal analysis approaches

We define causal network analysis as the process of inferring potential regulatory mechanisms by integrating experimental data with a PKN. We are particularly interested in identifying causal regulators whose activity may causally explain downstream changes observed in the data. The idea is to evaluate whether experimental observations are consistent with the interactions already captured within the prior knowledge.

**Figure 2:**
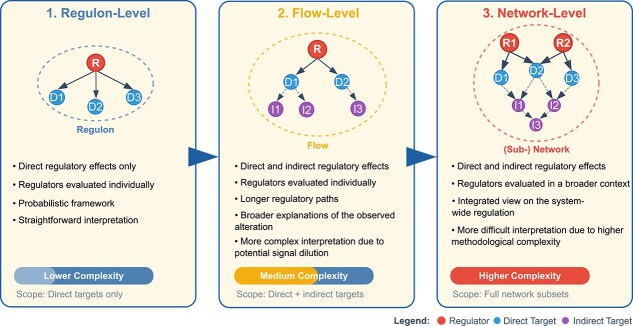
Comparative overview of three approaches to causal network analysis of omics data. This figure illustrates the three methodological categories for causal network analysis, showing increasing scope and complexity. Regulon-level analysis (left) examines individual regulators and their direct targets. Flow-level analysis (middle) traces multi-step signaling cascades from upstream regulators to downstream effectors. Network level analysis (right) considers system-wide regulatory interactions and crosstalk between multiple regulators. The legend indicates regulators (red), direct targets (blue), and indirect targets (purple). Alt text: Three network diagrams showing progression of causal analysis methods: regulon-level analysis depicting a single regulator (red) with direct targets (blue); flow-level analysis showing a signaling cascade from upstream regulator through intermediate nodes to downstream targets; network-level analysis displaying multiple interconnected regulators with overlapping targets and crosstalk.

The reviewed methods are grouped into three categories based on the extent to which PKNs are used: (i) *regulon-level* approaches analyze individual transcription factors and their direct targets in isolation; (ii) *flow-level* approaches trace multi-step signaling cascades from upstream regulators through intermediate nodes to downstream effectors; and (iii) *network-level* approaches consider system-wide interactions among multiple regulators, their targets, and regulatory crosstalk simultaneously (see overview in Fig. 2). A regulon refers to a regulator and its direct downstream targets; a flow includes a regulator and its direct and indirect downstream targets; a network here refers to a subnetwork within a larger molecular network. This categorization reflects a progression in methodological complexity and enables more meaningful comparisons.

Our qualitative comparative approach is necessitated by the heterogeneity in methodologies and applications, which precludes direct performance benchmarking. The methods target distinct research questions, making standardized performance metrics inappropriate.

### Regulon-level analysis

*Background* *:* given a set of bioentities with altered activity, one may be interested in identifying the direct upstream regulators responsible. For each candidate regulator in the PKN, the subnetwork of its direct downstream targets (its regulon) is identified. The observed directions of alterations are then compared with those expected from the prior knowledge. To formalize this, sign counts are summarized in a contingency table for each regulon (Supplementary Table S1).

*Methods* *:* one of the first causal reasoning approaches is *Whistle*, proposed by Catlett *et al.* [34], which evaluates the counts of matching (${\mathrm{n}}_{++},{n}_{--}$) and opposing (${n}_{+-},{n}_{-+}$) signs between predicted and observed directions of alterations. It assumes the probability of a pattern follows a binomial distribution:


$$prob=\left(\genfrac{}{}{0pt}{}{n_{++}+{n}_{+-}+{n}_{-+}+{n}_{--}}{n_{++}+{n}_{--}}\right){p}^{n_{++}+{n}_{--}}\cdotp{\left(1-p\right)}^{n_{+-}+{n}_{-+}}.$$

In this formula, *p* is the binomial parameter, which the authors suggest setting to 0.5, reflecting an equal chance of a correct or incorrect prediction. A drawback is that the resulting *P*-values are influenced by regulon size, potentially favoring larger regulons [34].

An alternative method, *CRE* (Causal Reasoning Engine), by Chindelevitch *et al.* [35], also uses these counts but evaluates probabilities using the hypergeometric distribution, which accounts for regulon size through the margins of the contingency table:


$$prob=\frac{\left(\genfrac{}{}{0pt}{}{q_{+}}{n_{++},{n}_{+-},{n}_{+0}}\right)\left(\genfrac{}{}{0pt}{}{q_{-}}{n_{-+},{n}_{--},{n}_{-0}}\right)\left(\genfrac{}{}{0pt}{}{q_0}{n_{0+},{n}_{0-},{n}_{00}}\right)}{\left(\genfrac{}{}{0pt}{}{N}{n_{+},{n}_{-},{n}_0}\right)},$$

where ${n}_{+0},{n}_{-0}$ and ${n}_{00}$ denote counts of non-differentially abundant bioentities; ${q}_{+},{q}_{-}$ and ${q}_0$ are the marginal column sums, and $N={q}_{+}+{q}_{-}+{q}_0.$

Next, *IURA* (QIAGEN Ingenuity Upstream Regulator Analysis), developed by Krämer *et al.* [36], incorporates edge weights from PKDBs like QBKB-HD. IURA integrates these weights into its statistical framework, calculating a z-score based on matching and opposing sign pairs:


$$ z(r)=\frac{\sum_{\nu \in \overset{\sim }{O}}{w}_R\left(r,\nu \right){s}_R\left(r,\nu \right){s}_D\left(\nu \right)}{\sqrt{\sum_{\nu \in \overset{\sim }{O}}{\left[{w}_R\left(r,\nu \right)\right]}^2}}, $$

where *r* is a candidate regulator, ${s}_R\left(r,\nu \right)$ is the predicted direction of regulation, ${s}_D\left(\nu \right)$ is the observed alteration sign, and ${w}_R\left(r,\nu \right)$ is the edge weight. The sum goes over direct downstream bioentities of *r* such that ${s}_R\left(r,\nu \right)\ne 0,{s}_D\left(\nu \right)\ne 0$. Under the null hypothesis, this z-score is approximately normally distributed.

So far, none of the discussed statistics account for unsigned regulatory relationships. To address this, each method incorporates an additional enrichment statistic (a one-sided Fisher’s exact test) to evaluate the overrepresentation of regulated targets within a regulon.

More recently, the *QS* (Quaternary Score) method by Fakhry *et al.* [37] integrates both signed and unsigned relationships into a single test statistic:


$$ QS={n}_{++}+{n}_{--}+{n}_{r+}+{n}_{r-}-\left({n}_{+-}+{n}_{-+}\right). $$

Here, ${n}_{r+}$ and ${n}_{r-}$ denote counts of bioentities with known influence but unknown regulation direction. A *P*-value is computed by summing the probabilities of all contingency tables with scores at least as large as the observed one, where the probability is calculated using a hypergeometric formula that accounts for the unsigned category.

Finally, *VIPER* (Virtual Inference of Protein Activity by Enriched Regulon Analysis**)**, introduced by Alvarez *et al.* [38], uses a rank-based approach similar to Gene Set Enrichment Analysis (GSEA) [39]. It incorporates continuous measures of differential expression, allowing it to weigh predictions more effectively and avoid arbitrary significance thresholds. VIPER computes a signed enrichment score for each putative regulator and tests its statistical significance.

All five methods not only assess statistical significance but also facilitate conclusions about whether a potential regulator is likely activated or inhibited. An overview is provided in Table 3.

**Table 3:** Overview of the key features of regulon-level analysis methods.

	**Unsigned edges**	**Weighted edges**	**Arbitrary input PKN**	**Code availability**	**Software reference**
Whistle	^a^	No	^b^	Yes, Java	https://github.com/Selventa/whistle
CRE	^a^	No	Yes	Yes, R	^c^
QS	Yes	No	Yes	Yes, R	http://bioconductor.org/packages/QuaternaryProd
IURA	^a^	Yes	No, only QBKB-HD	^d^No	https://digitalinsights.qiagen.com
VIPER	No	Yes	Yes	Yes, R	http://bioconductor.org/packages/viper

The columns “Unsigned edges” and “Weighted edges” indicate if the statistic for a method makes use of unsigned edges and edge weights. The column “Arbitrary input PKN” indicates if a method can be used with a user-specified PKN.

^a^Via an enrichment statistic;

^b^PKN should be provided in BEL (Biological Expression Language) format;

^c^Original code is available upon request to the authors and the method is also implemented within the QS package;

^d^IURA is implemented within the commercial QIAGEN Ingenuity Pathway Analysis software.

#### Prior applications

Regulon-level methods have demonstrated significant performance in recovering known perturbations. Whistle was applied to TNF exposure profiles and successfully identified known TNF signaling molecules [34]. CRE and QS were evaluated on perturbation signatures involving c-MYC and H-Ras, recovering either the perturbed regulator or a functionally related molecule [35, 37]. VIPER was tested in lymphoma cell line experiments where individual regulators were silenced, successfully identifying them as top inactivated proteins [38]. Furthermore, CRE was used to gain mechanistic insight into how a small molecule compound preserves T cell function [40], and VIPER helped investigate transcriptional changes in schizophrenia [41].

#### Strengths and limitations

Regulon-level analysis benefits from its straightforward focus on direct regulatory relationships, which facilitates interpretation. Additionally, these methods adopt a probabilistic framework, providing a measure of uncertainty for every candidate regulator. At the same time, the main limitation is its core assumption of treating regulons independently. In reality, regulons interact, and different regulators may control the same target genes. Disregarding these connections can lead to an oversimplified representation of complex regulatory networks.

### Flow-level analysis

#### Background

Identifying upstream regulators not directly connected to the observed changes requires tracing longer paths in the graph. A “flow” refers to a set of nodes in a causal graph that all trace back to a common upstream regulator. These methods require access to the complete PKN, making commercial databases with limited accessibility unsuitable. A fully accessible resource such as SIGNOR is a suitable alternative.

#### Methods

One of the first flow-level analysis methods is *CARNIVAL* (CAusal Reasoning pipeline for Network identification using Integer VALue programming), proposed by Liu *et al.* [42]. It casts flow derivation as an integer linear programming optimization problem, formulated to find a flow in a parsimonious manner:


$$ mi{n}_{V_{act},{V}_{inh}}\sum_{\nu \in V}\left|{d}_{\nu}\right|\left(1-{\sigma}_{\nu}\left({V}_{act}\left(\nu \right)-{V}_{inh}\left(\nu \right)\right)\right)+\mathrm{\beta} \cdotp \mathcal{R}, $$

where ${d}_{\nu }$ is the measurement for node $\nu$; ${\mathrm{\sigma}}_{\nu }={{sign}}\left({d}_{\nu}\right);$  $\mathrm{\beta}$ is a regularization coefficient, and $\mathcal{R}$ is a regularization term that penalizes the use of additional nodes or edges; ${V}_{act}\left(\nu \right),{V}_{inh}\left(\nu \right)$ are binary indicators representing activation and inhibition of node $\nu$, respectively. CARNIVAL is available in two modes: standard (StdCARNIVAL), which uses prior knowledge of perturbed regulators as input, and inverse (InvCARNIVAL), which jointly infers both the upstream regulators and the signaling pathways leading to the observed downstream alterations.

The ideas of CARNIVAL were extended in *SignalingProfiler* by Massacci *et al.* [43] and further developed by Venafra *et al.* [44]. Instead of treating all downstream targets as a single group, this framework categorizes them into distinct functional groups (kinases, phosphatases, transcription factors, etc.) arranged in hierarchical layers. It then runs StdCARNIVAL sequentially between layers to construct an integrated flow. Activities of intermediate signaling proteins and transcription factors are estimated using VIPER [38]. This multi-layered design improves the biological relevance and relaxes the restrictive tree structure of the original CARNIVAL.

#### Prior applications

CARNIVAL was originally validated in a study of IgA nephropathy [42]. Analysis of the resulting flows identified pathways known to be associated with the disease, and experimental validation of key identified nodes of interest (RhoA and β-catenin) was consistent with CARNIVAL’s predictions. SignalingProfiler was developed to study FLT3-ITD-positive acute myeloid leukemia, revealing distinct modes of regulation involving the protein kinase WEE1 between subtypes [43]. Pharmacological inhibition of WEE1 was then shown to restore therapy sensitivity, highlighting WEE1 as a potential therapeutic target.

#### Strengths and limitations

Flow-level analysis provides a broad view of mechanisms behind observed alterations by tracing longer signaling paths. These methods are also robust to noise as they typically operate on inferred node activities (e.g. through regulon-level analysis) rather than raw omics data. However, a main limitation is that signal propagation over longer paths can make interpretation more uncertain. Furthermore, these methods do not operate within a probabilistic framework and do not provide statistical confidence measures for the inferred flows.

### Network-level analysis

#### Background

Neither regulons nor flows may provide a complete picture, since interactions between high-level regulators are not fully captured. To obtain a more comprehensive view, network-level methods consider interactions across the entire network of regulators and targets.

#### Methods

A recent network-level framework is *CORNETO* (Constrained Optimisation for the Recovery of NETworks from Omics) by Rodriguez-Mier *et al.* [45], which includes a multi-sample CARNIVAL component. Potential regulators and their condition-dependent activity changes are provided as inputs for each sample. A key feature is that the same PKN topology is used across all samples, and the optimization problem favors edges that are reused across samples, thus identifying common signal propagation paths:


\begin{align*} {min}\ \left(\frac{1}{n}\sum_{i=1}^n\sum_{\nu \in V}\left|{d}_{\nu}^i\right|\left(1-{\sigma}_{\nu}^i\left({V}_{act}^i\left(\nu \right)-{V}_{inh}^i\left(\nu \right)\right)\right)\right.\\\left.\phantom{\sum_{i=1}^n}+\#\left\{ Total\ number\ of\ edges\ used\right\}\right), \end{align*}

where the minimization is performed over all ${V}_{act}^i\left(\nu \right)$ and ${V}_{inh}^i\left(\nu \right)$, denoting activation and inhibition indicators for all samples *i* from 1 to *n*. The regularization term penalizes the total number of edges used, thereby favoring edges that are reused across samples. This reflects a preference for identifying common signal propagation paths originating from the perturbed nodes.

*TopoNPA*, proposed by Martin *et al.* [46], quantifies the consistency between the observed downstream alteration pattern and a PKN. It employs a two-layer model (observed transcript layer and unobserved functional layer). An optimization problem is formulated to infer alteration values *f* in the functional layer:


$$ mi{n}_{f\in{l}^2(V)}\sum_{x\to y}{\left(f(x)+{{sign}}\left(x\to y\right)f(y)\right)}^2\cdotp w\left(x,y\right), $$

such that ${\left.f\right|}_{V_0}=\mathrm{\beta}$.

In this expression, the sum is taken over all directed edges $\left(x\to y\right)$; $f(x)$ denotes the inferred alteration value of node $x;{V}_0$ is the subset of nodes in the transcript layer; $\beta$ is a vector of log-fold changes in the transcript layer; and $w\left(x,y\right)$ is the weight of the edge between $x$ and $y$ (see Supplementary Material, Note S1). An analytical solution to this optimization problem can be derived by reformulating it in terms of the adjacency matrix of the graph (see Supplementary Material, Note S2) and solving the resulting matrix equation.

The *NLBayes* method by Arriojas *et al.* [47] builds on the regulon-level approach by incorporating information about interactions between regulons that share common targets (see also Supplementary Fig. S1). It employs a Bayesian framework that distinguishes between the true and observed states of nodes, allowing it to account for potential errors in differential analysis. Inference is performed by maximizing the likelihood function through a sampling procedure.

Finally, *GRNOptR* (Gene Regulatory Network Inference Using Optimization), introduced by Zickenrott *et al.* [48] and further improved by Hartmann and Ali (https://git-r3lab.uni.lu/CBG/GRNOptR) follows a two-step procedure. The first step contextualizes the PKN by pruning it to a subnetwork i.e. consistent with observed alteration patterns. This is done by solving a constrained maximization problem to preserve as many logically consistent edges as possible. The second step identifies candidate “perturbagens”, i.e. nodes whose perturbation could potentially revert the observed alteration pattern. This is assessed by propagating a flipped activity state for a candidate node through the network and counting how many downstream nodes switch their state (see also Supplementary Fig. S2). GRNOptR is the only reviewed method that infers the signs of previously unsigned edges.

A summary of flow- and network-level methods is provided in Table 4.

**Table 4:** Summary of flow- and network-level methods for causal analysis. The columns show the names and publication years of the computational approaches, the method type (Flow = flow-level analysis, NW = network-level analysis), the output types, the algorithmic foundations (brief simplified description), and links to the software implementations.

**Method**	**Level**	**Output**	**Algorithm (simplified)**	**Software with reference**
**CARNIVAL** (2019)	Flow	Set of flows	Integer linear programming	R: http://bioconductor.org/packages/CARNIVAL
**SignalingProfiler** (2024)	Flow	Set of flows through predefined functional layers	VIPER to identify dysregulated nodes + multiple CARNIVALs	R: https://github.com/SaccoPerfettoLab/SignalingProfiler
**CORNETO** (2025)	Flow/ NW	Set of flows / a parsimonious NW explaining alteration in multiple samples	Integer linear programming	Python: https://github.com/saezlab/corneto
**TopoNPA** (2014)	NW	Concordance score between a subNW and observed alteration; set of contribution scores per regulator	Analytical calculation in matrix algebra	R: [49] Python: https://github.com/mikethenut/perturbationx
**NLBayes** (2023)	NW	List of activated regulators	Gibbs sampling using Markov blankets	R: https://github.com/umbibio/nlbayes-r Python: https://github.com/umbibio/nlbayes-python
**GRNOptR** (2016)	NW	A subnetwork of direct connections between differentially expressed genes; perturbagen-score pairs	Constraint based optimization	R: https://git-r3lab.uni.lu/CBG/GRNOptR

*Prior applications* *:* TopoNPA was used to evaluate a regulatory network model for cardiotoxicity in zebrafish, where it correctly identified perturbations in four out of five experimental conditions [50]. GRNOptR was applied to investigate sphingolipid metabolism in Alzheimer’s disease by Giovagnoni *et al.* [51]. It was used to build a consistent gene regulatory network, which then served to identify several candidate regulatory genes (CAV1, SPHK1, and SELPLG) as key perturbagens. These findings aligned well with previous literature, suggesting a causal role for these genes in the observed alterations [52–54].

**Table 5:** Unified comparison of causal analysis methods.

**Method (Approach)**	**Omics Data Types** ^c^	**PKDB Requirements**	**Framework**	**Interpretability**	**Software maturity**	**Advantages**	**Limitations**
**Whistle** (Regulon-level)	Omics-agnostic	A set of regulons encoded in BEL^a^ suffices	Binomial test (P)	High	Moderate (Outdated)	Simple, interpretable	Biased towards bigger regulons; ignores effect sizes and unsigned interactions
**CRE** (Regulon-level)	Omics-agnostic	A set of regulons suffices	Hypergeometric test (P)	High	Mature (Bioconductor maintained)	Accounts for regulon sizes	Ignores effect sizes; main statistic ignores unsigned interactions
**QS** (Regulon-level)	Omics-agnostic	A set of regulons suffices	Hypergeometric test (P)	High	Mature (Bioconductor maintained)	Accounts for regulon sizes; incorporates unsigned edges	Ignores effect sizes
**IURA** (Regulon-level)	Omics-agnostic	QBKB-HD (commercial QIAGEN database)	Z-score (P)	High	Mature (enterprise support)	Incorporates edge weights	Commercial software; main statistic ignores regulon sizes, effect sizes and unsigned interactions
**VIPER** (Regulon-level)	Omics-agnostic	A set of regulons suffices	GSEA-like enrichment (P)	High	Mature (widely adopted)	Incorporates effect sizes and weighted edges; accounts for regulon sizes	Ignores unsigned interactions
**CARNIVAL** (Flow-level)	Omics-agnostic	Fully downloadable^b^	Integer linear programming (nonP)	Moderate: multi-step signalling	Mature (Bioconductor maintained)	Traces multi-step signaling cascades	Requires optimization solver
**SignalingProfiler** (Flow-level)	Transcriptomics, (phospho)proteomics	Fully downloadable^b^	Hybrid (VIPER + multiple CARNIVALs) (nonP)	Moderate-to-high: layered (still multi-step) signaling	Moderate (GitHub only)	Supports study-defined functional layers	Complex setup, requires optimization solver
**CORNETO** (Flow/NW-level)	Omics-agnostic	Fully downloadable^b^	Integer linear programming (nonP)	Moderate: multi-step signalling	Moderate (early stage)	Designed for multi-sample analysis	Requires optimization solver
**TopoNPA** (NW-level)	Omics-agnostic	Fully downloadable^b^	Analytically solvable optimization (P)	Moderate-to-high: optimization-inferred scores	Moderate (GitHub only)	Incorporates effect sizes; has analytical solution	No edges between observed nodes
**NLBayes** (NW-level)	Transcriptomics	A set of regulons suffices	Bayesian graphical model (P)	Moderate-to-high: sampling-based inference	Moderate (early stage)	Captures regulon crosstalk	Sampling-based inference; “inhibition” for regulators not inferred
**GRNOptR** (NW-level)	Omics-agnostic	Fully downloadable^b^	Constraint-based optimization (nonP)	Moderate: multi-step signalling	Moderate (GitLab only)	Infers edge signs	Ignores effect sizes

Comparison by approach, supported omics, prior-knowledge requirements, methodological framework, interpretability (qualitative), software maturity (indicative), and key advantages/limitations.

*Abbreviations and terms:* In “Framework” (P) indicates probabilistic methods, (nonP) – non-probabilistic (e.g. linear programming). **PKN**—prior-knowledge network (directed regulator→target graph); **PKDB** – prior-knowledge database (source of PKNs);

^a^ **BEL**—Biological Expression Language; **GSEA**—Gene Set Enrichment Analysis (rank-based enrichment); **QBKB-HD**—QIAGEN Knowledge Base;

^b^ **“Fully downloadable PKN”** is a publicly available network offered for bulk download;

^c^ **Omics-agnostic**—method can operate on any assay provided the PKN nodes match the measured entities and edges encode the expected effect.

*Strengths and limitations* *:* network-level approaches offer the most advanced level of data integration, potentially capturing complex system-wide patterns. Additionally, probabilistic frameworks of TopoNPA and NLBayes provide statistical confidence measures, addressing a limitation of flow-level methods. GRNOptR offers the unique benefit of inferring signs of previously unsigned interactions. Regarding limitations, as methodology becomes more complex, interpretation becomes more challenging. Some methods still lack probabilistic confidence measures, and like flow-level methods, rely on signal propagation across long paths. A specific limitation for GRNOptR is that its pruning procedure treats inputs as binary (up or down), so the most strongly perturbed bioentities may not be adequately reflected.

### Practical guidance for method selection

**Figure 3:**
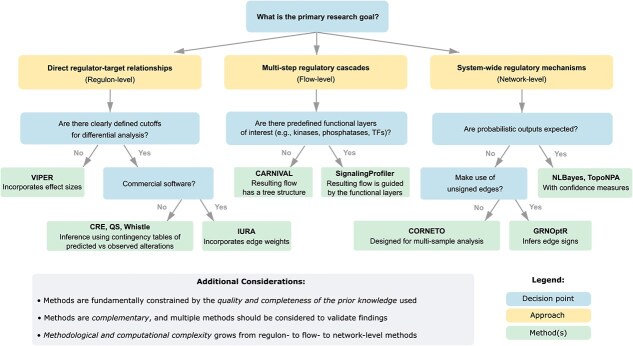
Decision tree for selecting appropriate causal network analysis methods based on research goals and data characteristics. The workflow guides researchers from their primary research question through three main analytical approaches: regulon-level analysis for direct regulator-target relationships, flow-level analysis for multi-step regulatory cascades, and network-level analysis for system-wide regulatory mechanisms. Each approach branches into specific methods based on key decision points: availability of defined cutoffs for differential analysis, presence of predefined functional layers, and whether probabilistic outputs are expected. Methods increase in complexity from left to right. Alt text: Flowchart showing method selection for causal network analysis. From top question “What is the primary research goal?” three paths branch to: regulon-level (left, yellow box), flow-level (middle, yellow box), and network-level (right, yellow box). Each path contains decision points (blue boxes) leading to recommended methods (green boxes). Left path asks about differential analysis cutoffs, leading to VIPER or commercial software, then to CRE/QS/whistle or IURA. Middle path asks about functional layers, leading to CARNIVAL or SignalingProfiler. Right path asks about probabilistic outputs, leading to unsigned edges methods or NLBayes/TopoNPA, then to CORNETO or GRNOptR. Bottom section lists three key considerations about prior knowledge quality, complementary methods, and increasing computational complexity.

This subsection offers a brief, practical guide to method selection. We start from the premise that questions addressed by causal network analysis fall into the three approaches discussed earlier: (i) regulon-level for identifying direct upstream regulators; (ii) flow-level for tracing longer regulatory cascades; (iii) network-level for identifying system-wide regulatory mechanisms. Once the goal is set, method selection is refined by several decision criteria: inputs from differential analysis (gene list or ranking), any focus on specific functional layers, preferred methodological framework (probabilistic versus optimization/integer programming), and other practical constraints (e.g. access to the commercial software, use of unsigned edges). The aim is not to prescribe a single “best” tool but to make trade-offs explicit and to encourage complementary use where appropriate. The decision guide is shown in Fig. 3.

In addition to the guideline, a unified comparison of the reviewed methods and corresponding software is provided in Table 5, summarizing data requirements, PKDB compatibility, methodological framework, interpretability, software maturity as well as key advantages and limitations.

## Conclusion

Causal network analysis is an important computational framework that facilitates the inference of regulators and mechanisms underlying observed alterations. Our classification of methods into regulon-level, flow-level, and network-level approaches reveals distinct strengths and limitations based on the extent of prior knowledge integration. Regulon-level methods offer interpretability and statistical rigor but may oversimplify regulatory interactions. Flow-level approaches capture broader regulatory cascades but can sacrifice interpretability. Network-level methods provide the most comprehensive view while introducing greater complexity.

While quantitative benchmarking is valuable, causal network analysis presents challenges that necessitate a qualitative approach, as adopted in this review. The methods discussed are designed to yield different biological insights rather than representing competing solutions to the same problem. This diversity, combined with the absence of comprehensive ground truth datasets, makes standardized benchmarking potentially misleading. Our qualitative comparison emphasizes the complementary nature of these approaches.

Despite their utility, these methods share a fundamental limitation: they are constrained by the quality and completeness of the prior knowledge used. Missing regulators in knowledge bases cannot be identified. Moreover, PKDBs often exhibit systematic curation/literature biases (e.g. overrepresentation of cancer/immunology) that can inflate apparent support in heavily annotated domains and pull focus away elsewhere. This emphasizes the critical importance of continuously updating biological interaction databases and calls for cautious interpretation when prior knowledge is incomplete. Promising future directions include developing hybrid approaches that combine causal network analysis with data-driven causal inference methods to identify novel regulatory relationships. While this review centers on network-based reasoning, it is important to note that data-driven causal inference methods offer complementary capabilities for molecular analysis. Recent bioinformatics applications include MRPC for molecular causal graphs (this method, in particular, is suited to integrating multiple omics layers) [55], hybrid Bayesian/optimization approaches such as GOBNILP [56] and PC-with-NOTEARS [57], and emerging deep-learning methods like GFlowNet-based causal GRN discovery [58] and SLIVER [59].

Future developments of causal network analysis will likely be driven by the increasing availability of multi-omics data and improved prior biological knowledge [60, 61]. Next-generation methods should jointly analyze multiple molecular layers while accounting for their specific properties. Key methodological priorities include providing probabilistic confidence scores, ensuring robustness to observational noise, and avoiding overextension of signal propagation to distant, unobserved nodes. In summary, causal network analysis represents a mature and valuable approach for understanding disease mechanisms. Continued progress will improve our ability to generate meaningful biological hypotheses and support evidence-based biomedical research.

**Key Points**

- Causal network analysis methods integrate experimental omics data with prior knowledge databases and offer a powerful approach to identify upstream regulators and mechanistic explanations for observed molecular changes, going beyond simple correlation-based analyses to infer potential causation.
- Methods can be grouped into three approaches based on their use of prior knowledge: regulon-level methods analyze direct regulator-target relationships, flow-level methods trace multi-step signaling cascades, and network-level methods consider system-wide regulatory interactions and crosstalk.
- Regulon-level methods provide interpretable results with statistical confidence measures but oversimplify complex regulatory networks, while flow- and network-level approaches capture broader mechanistic views at the cost of increased complexity (both methodological and computational) and sometimes reduced interpretability.
- Causal network analysis is bounded by the completeness and quality of prior-knowledge databases; in an inherently incomplete knowledge setting, results require cautious interpretation.

## Supplementary Material

Supplementary_review_causal_network_analysis_of_omics_data_bbaf654

## Data Availability

Data sharing is not applicable to this article as no datasets were generated or analyzed during the current study. The review is based on manual review and synthesis of previously published literature cited in the references.
