# Supplementary material for: Causal network analysis of omics data using prior knowledge databases
Source: Brief Bioinform. 2025 Dec 5;26(6):bbaf654. doi: 10.1093/bib/bbaf654 (PMC12703490; doi:10.1093/bib/bbaf654)
Supplement: Supplementary_review_causal_network_analysis_of_omics_data_bbaf654 [file supplementary_review_causal_network_analysis_of_omics_data_bbaf654.pdf]

Supplementary material for
"Causal network analysis of omics data using prior knowledge databases"

Gleb Svinin[1] and Enrico Glaab[1]

[1] Biomedical Data Science Group, Luxembourg Centre for Systems Biomedicine (LCSB), University of Luxembourg, Esch-sur-Alzette, Luxembourg

Corresponding author:

Enrico Glaab, PhD
Luxembourg Centre for Systems Biomedicine
University of Luxembourg
Tel. +352 621 621 6186
E-mail: enrico.glaab@uni.lu

Supplementary Information – Overview

1. **Supplementary Table S1: Schematic contingency table for a regulon** p. 2
2. **Supplementary Figure S1: Two exemplary regulons illustrating the NLBayes model** p. 3
3. **Supplementary Figure S2: Exemplary pipeline of GRNOptR network pruning** p. 4
4. **Supplementary Note S1: Causal graph** p. 5
5. **Supplementary Note S2: Adjacency matrix of the graph** p. 5
6. **Supplementary Note S3: Consistency of adjacent signed nodes** p. 6

	Observed +	Observed -	Observed 0	Total
**Predicted +**	n++	n+-	n+0	q+
**Predicted -**	n-+	n--	n-0	q-
**Predicted r**	nr+	nr-	nr0	qr
**Predicted 0**	n0+	n0-	n00	q0
**Total**	n+	n-	n0	

Supplementary Table S1: Contingency table structure for regulon-level causal analysis. The table shows the cross-tabulation of predicted versus observed alteration states for bioentities within a regulon. Green cells represent correct predictions (matching signs), red cells represent incorrect predictions (opposite signs), yellow cells represent unsigned interactions (used by specific methods like QS), and gray cells represent unobserved interactions (excluded from analysis). Marginal totals (q+, q-, qr, q0 and n+, n-, n0) are used for statistical significance testing in regulon-level methods.

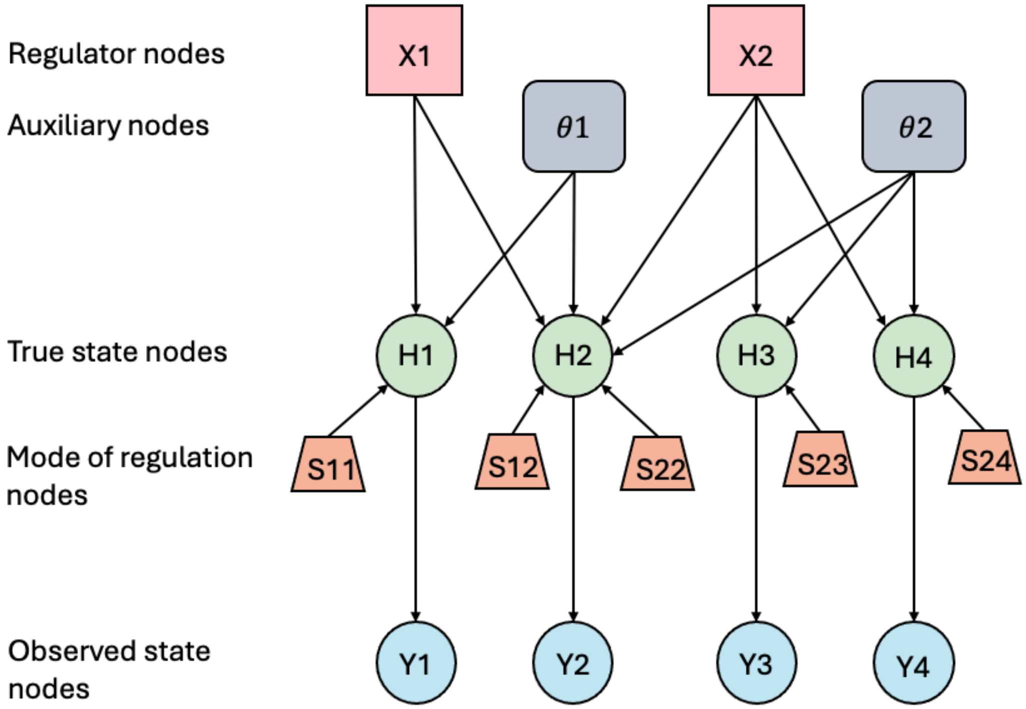

Supplementary Figure S1: Bayesian network structure underlying the NLBayes model. The diagram shows two interconnected regulons controlled by regulators X1 and X2, each associated with noise variables η1 and η2. Random variables model the true activity states (H1–H4) conditional on regulator activity. Variables S11–S24 represent the modes of regulation (activating, inhibiting, or unknown). Regulator X1 controls bioentities H1 and H2 through regulatory modes S11 and S12, respectively. Regulator X2 controls bioentities H2, H3, and H4 through regulatory modes S22, S23, and S24, respectively. Note that H2 is regulated by both X1 and X2, illustrating the model's ability to handle overlapping regulons.

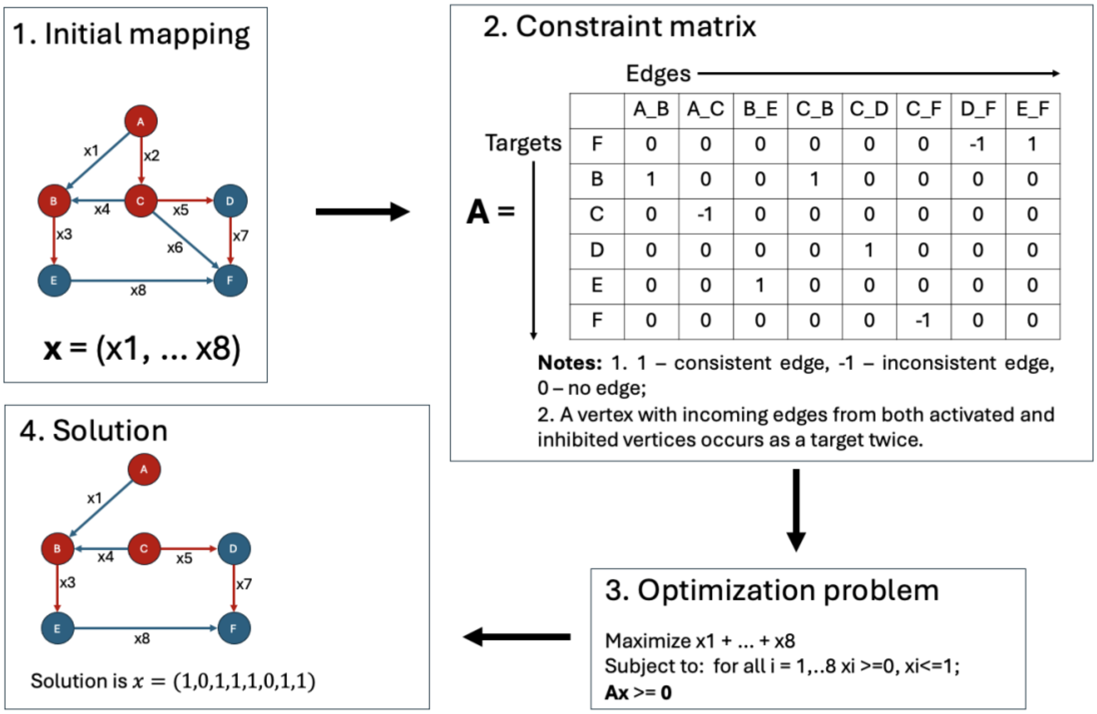

Supplementary Figure S2: GRNOptR network pruning workflow example. The figure illustrates the constraint-based optimization process for constructing consistent subnetworks from prior knowledge networks. 1) Differential expression data is mapped onto the PKN. Nodes are colored according to their expression changes: red for decreased, blue for increased expression. Edges represent regulatory interactions: red for inhibition, blue for activation. Each edge is assigned a variable weight x1,...x8, initially undefined. 2) The network is converted into a constraint matrix, where each row corresponds to a target node and each column to a specific edge. Positive entries indicate consistency between the source and target node, negative entries indicate inconsistency, and zeros denote non-incident edges. 3) A constrained maximization problem is solved to maximize the sum x1+..+x8. This reflects the goal of preserving as many edges as possible while ensuring that the state of each node remains consistent with its incoming edges. 4) The output is a pruned subnetwork along with the solution of the optimization problem, indicating which edges should be retained.

Supplementary Note S1: Definition of a causal graph

A causal graph G is formally defined as a pair G = (V, E), where:

1. V is a finite set of nodes (vertices), each associated with a sign ∈ {+, −, 0} representing increased, decreased, or unaffected activity states, respectively.
2. $E \subseteq V \times V$ is a set of directed edges representing causal relationships, where each edge (u, v) ∈ E denotes a causal influence from source node u to target v. Each edge is assigned a sign ∈ {+, −, 0} corresponding to activation, inhibition, or unknown regulation, respectively.

Supplementary Note S2: Adjacency matrix representation of the graph. The adjacency matrix A of the graph (V, E) is a square matrix of size $|V| \times |V|$, where each entry A_{ij} is defined as follows:

1. $A_{ij} = 1$ if node i activates node j,
2. $A_{ij} = -1$ if node i inhibits node j,
3. $A_{ij} = 0$ if there is no edge from i to j.

This matrix representation enables efficient computational operations for network-based algorithms such as those implemented in TopoNPA and other network-level analysis methods.

Supplementary Note S3: Consistency rules for signed nodes. Two adjacent signed nodes are considered consistent if the alteration state of the target node matches the state predicted according to the source node and regulatory relationship. Formally, the nodes are consistent when:

$$\text{sign}(\text{target}) = \text{sign}(\text{source}) \times \text{sign}(\text{edge})$$

where sign(target) and sign(source) $\in \{+1, -1\}$ represent the alteration states, and sign(edge) $\in \{+1, -1\}$ represents the regulatory relationship (activation or inhibition). This consistency principle forms the basis for constraint-based optimization methods such as GRNOptR.
